# Rates of gene conversions between *Escherichia coli* ribosomal operons

**DOI:** 10.1093/g3journal/jkaa002

**Published:** 2020-11-11

**Authors:** Isaac Gifford, Aurko Dasgupta, Jeffrey E Barrick

**Affiliations:** Department of Molecular Biosciences, Center for Systems and Synthetic Biology, The University of Texas at Austin, Austin, TX 78712, USA

**Keywords:** gene conversion, mutation rate, concerted evolution, mutation accumulation experiment, ribosomal RNA, 16S sequencing

## Abstract

Due to their universal presence and high sequence conservation, ribosomal RNA (rRNA) sequences are used widely in phylogenetics for inferring evolutionary relationships between microbes and in metagenomics for analyzing the composition of microbial communities. Most microbial genomes encode multiple copies of rRNA genes to supply cells with sufficient capacity for protein synthesis. These copies typically undergo concerted evolution that keeps their sequences identical, or nearly so, due to gene conversion, a type of intragenomic recombination that changes one copy of a homologous sequence to exactly match another. Widely varying rates of rRNA gene conversion have previously been estimated by comparative genomics methods and using genetic reporter assays. To more directly measure rates of rRNA intragenomic recombination, we sequenced the seven *Escherichia coli* rRNA operons in 15 lineages that were evolved for ∼13,750 generations with frequent single-cell bottlenecks that reduce the effects of selection. We identified 38 gene conversion events and estimated an overall rate of intragenomic recombination within the 16S and 23S genes between rRNA copies of 3.6 × 10^−4^ per genome per generation or 8.6 × 10^−6^ per rRNA operon per homologous donor operon per generation. This rate varied only slightly from random expectations at different sites within the rRNA genes and between rRNA operons located at different positions in the genome. Our accurate estimate of the rate of rRNA gene conversions fills a gap in our quantitative understanding of how ribosomal sequences and other multicopy elements diversify and homogenize during microbial genome evolution.

## Introduction

Ribosomes perform some of the most highly conserved chemistry in cells—the translation of messenger RNAs into proteins—and ribosomal RNA (rRNA) genes are the most conserved genes across all domains of life (Isenbarger *et al.* 2008). Because of their low rate of evolution and ubiquity across taxonomic divisions, the nucleotide sequence of the small subunit RNA, known in bacteria and archaea as the 16S and in eukaryotes as the 18S, is often used to measure divergence between distantly related species (Woese 1987) and is therefore one basis for reconstructing the “Tree of Life” (Fournier and Gogarten 2010). Small ribosomal subunit amplicon sequencing is also commonly used to estimate the representation of different species in environmental and host-associated microbial communities (Okuda *et al.* 2012; Langille *et al.* 2013). 

The need for new protein synthesis is often the dominant factor limiting cellular replication (Scott *et al.* 2010; Ceroni *et al.* 2015), and ribosomes can account for up to 20% of the dry mass of rapidly dividing bacterial cells (Weider *et al.* 2005). To meet this high demand, most bacterial genomes encode multiple rRNA operons (Roller *et al.* 2016), and rRNAs are transcribed from highly active promoters (Paul *et al.* 2004). Bacterial species capable of more rapid growth generally have more rRNA operon copies in their genomes (Klappenbach *et al.* 2000; Roller *et al.* 2016), and deleting some of these operons generally reduces the maximum growth rate that a bacterial strain can sustain (Asai *et al.* 1999, Stevenson and Schmidt 2004).

rRNA genes and the intergenic regions between them tend to have extremely similar, if not identical, sequences within a single organism. This is thought to be due to a high rate of gene conversion, a type of non-reciprocal intragenomic homologous recombination between highly similar sequences that converts one into a precise copy of the other (Gürtler 1999; Liao 2000). In bacteria, gene conversions occur through RecA-dependent recombination (Prammananan *et al.* 1999) and can occur with or without crossing over (Santoyo and Romero 2005). Gene conversions can be either allelic, within multiple copies of the same gene, or ectopic, between sufficiently similar genes (Galtier 2001). Allelic gene conversions can lead to concerted evolution of multicopy genes when they occur at a sufficient rate that new mutations appearing in one copy are likely to be either reverted or propagated to all of the other homologous loci (Liao 1999, 2000; Gogarten *et al.* 2002). As they homogenize multicopy genes, allelic gene conversions have been proposed to be important for maintaining the ability of the cell to interchangeably use all copies of these genes for assembly of complex, multiple-subunit structures, like ribosomes (Liao 1999).

Accurate estimates of gene conversion rates are necessary to understand how they interact with other mutational processes during genome evolution. However, current estimates of the frequencies of gene conversions between *Escherichia coli* rRNA operons range over many orders of magnitude (Harvey and Hill 1990; Hashimoto *et al.* 2003). These studies, like many others looking at gene conversion, rely on reporter assays in which a single type of gene conversion is observed because it leads to a new phenotype that has a strong effect on cellular fitness (restored growth rate or resistance to a toxic metabolite, respectively). In such cases, viewing these frequencies through the prism of natural selection skews the inferred rates of gene conversions. By contrast, microbial mutation accumulation (MA) experiments propagate populations for many generations through single-cell bottlenecks to eliminate selection against all but the most deleterious mutations (Halligan and Keightley 2009). Mutation rates can be accurately determined from MA experiments simply by counting how many genomic changes of different types appear over time in replicate lineages (Foster *et al.* 2015).

We previously analyzed mutation rates in an MA experiment in which independent lineages of *E. coli* B were propagated for 550 days (Kibota and Lynch 1996; Tenaillon *et al.* 2016). Due to limitations of short-read resequencing data, we could not measure the rates of gene conversions in rRNA operons with that data. Here, we sequenced heterologous sites in all seven copies of the rRNA operon in 15 endpoint strains from this MA experiment. We use the observed changes to estimate an overall rate for gene conversions between highly homologous sequences and examine whether this rate varies substantially between different copies of the rRNA genes and sites within them.

## Materials and methods

### rRNA operon sequencing

The MA experiment has been described previously (Kibota and Lynch 1996; Tenaillon *et al.* 2016). Briefly, MA lineages were started from a 2000-generation clone isolated from a long-term evolution experiment (REL1206) that differs by a few mutations from *E. coli* B REL606 (Jeong *et al.* 2009). The MA experiment consisted of daily transfers in which a randomly selected colony was streaked out on Davis minimal medium agar supplemented with 200 μg/ml glucose. An estimated 13,750 generations elapsed over the course of the 550-day experiment (Tenaillon *et al.* 2016). We divided each of the seven *E. coli* rRNA operons into two PCR amplicons, one covering the *rrs* gene (16S) and the other including the *rrl* gene (23S) and the first downstream *rrf* gene copy (5S). PCR products for REL1206 and endpoint clones from 15 MA lineages were sequenced with the primers used for amplification and additional internal primers (Supplementary Table S1) to cover all heterologous rRNA sites. Sequencing was carried out at the UT Austin DNA Sequencing Facility on an ABI 3730xl DNA Analyzer (Thermo Fisher).

### Gene conversion inference

Trace files from PCR amplicon sequencing were aligned to the LTEE ancestor (REL 606) using Geneious 6. We found that REL1206 differed from REL606 by a single gene conversion: the 23S–5S spacer in the *rrnD* operon was converted to the *rrnAEH* type. Gene conversions in each evolved strain were identified manually by comparing the aligned PCR amplicon sequences to those of REL1206. In all instances in which a particular rRNA copy in the evolved strain differed from the corresponding copy in REL1206, the changes exactly matched at least one of the other rRNA copies. We inferred the properties of the most parsimonious set of gene conversions that could lead to these changes as follows. First, we determined which rRNA copies could have been donors for all bases changed by a given conversion. Next, we set the minimum conversion size as the length from the first changed base to the last changed base. Finally, we set the maximum conversion size as the largest 16S or 23S region from any of the donors that would only result in the observed changes. That is, we extended both ends through matching donor sequences until encountering a difference in all possible donor copies or the end of the rRNA subunit gene.

### Gene conversion rate analysis

The number of mutational events of a given type that occur in each MA experiment lineage is expected to follow a Poisson distribution with a time basis of 13,750 generations. However, unlike the case for calculating base substitution rates (Wielgoss *et al.* 2011; Foster *et al.* 2015), we cannot directly use the observed counts of changed rRNA sequences to calculate the gene conversion rate for two reasons. First, many rRNA gene conversion events will be undetectable because they occur between two sequences that are already identical. Second, an earlier gene conversion event may be hidden or reverted by a later event that covers the same bases. In order to account for both sources of unobservable events, we implemented a bootstrap resampling procedure. Input files and a Python script for performing this analysis are provided in Supplementary File S1.

The resampling procedure begins with a sequence alignment of the seven copies from the REL1206 genome of the rRNA region being analyzed that we created using MUSCLE v3.8.31 (Edgar 2004). We then generate sets of simulated conversions by selecting random sizes, locations that place these regions entirely within the rRNA subunit or spacer being analyzed, and donor and recipient operons. Simulated conversion sizes are determined by choosing a random event from the set of experimentally observed gene conversions in the rRNA region being analyzed, with replacement, and then picking a random size between the minimum and maximum sizes inferred for that conversion. A minimum size of 50 bp is enforced for small conversions because this is the required size for efficient RecA-mediated homologous recombination in *E. coli* (Singer *et al.* 1982; Watt *et al.* 1985). Using larger minimum conversion sizes of 100–300 bp did not appreciably alter our results. Conversions within the 23S–5S rRNA spacer were only considered to have one possible size, encompassing the entire spacer, because there is very little sequence homology between the two different ancestral rRNA operon alleles within this region and no partial spacer conversions were observed in the MA experiment.

To estimate gene conversion rates, we performed sets of 10,000 replicate simulations in which we drew a total number of gene conversion events for each of 15 simulated MA lineages from the Poisson distribution with a candidate rate according to the resampling procedure. Within each replicate and lineage, we applied the simulated conversions by copying the portion of the rRNA sequence that each one covered from the donor to the recipient operon. The final mutated sequences, after applying all conversions, were compared to the ancestral sequences to determine the smallest number of gene conversion events required to produce the observed sequence changes. This number of observable gene conversions is almost always less than the true number of simulated conversions. The maximum likelihood estimate of the *E. coli* gene conversion rate per genome was determined as the rate with the highest probability of resulting in exactly the same number of observable gene conversions as the MA experiment. The 95% confidence interval on this estimate was determined by finding a lower rate with a 2.5% tail probability of resulting in the number of observed gene conversions or more and a greater rate with a 2.5% tail probability of resulting in the observed number or fewer.

To understand variation in the rates of gene conversions between different rRNA operon copies and within different portions of their sequences, we performed 10,000 replicates of the resampling procedure in which we continued to draw new conversions until there were exactly as many observable conversions as were empirically observed. This allowed us to estimate 95% confidence intervals on the number of each type of change by taking the corresponding 2.5% and 97.5% quantiles from the 10,000 sets of resampled gene conversions. Two-tailed *P*-values for tests of whether the number of conversions experimentally observed within each operon or at each site was unexpected relative to the simulated null model were calculated as twice the proportion of resampled replicates with values as or more extreme than the observed conversion numbers and adjusted for multiple testing with a Bonferroni correction.

### Changes in rRNA sequence identity

Concatenated 16S and 23S subunit gene sequences were used to compare the homogeneity of rRNA operons within each MA endpoint clone and within the ancestor strain. Average percent sequence identity values were calculated as described by May (2004) using alignment length as the denominator for each pairwise combination of rRNA operons within each genome.

### Data availability

*Escherichia coli* strains are available upon request. Supplemental material available at figshare: https://doi.org/10.25387/g3.13143599. Supplementary Table S1 contains primer sequences. Supplementary Table S2 describes the gene conversions observed in the MA experiment. Supplementary File S1 contains the input files and Python script used to estimate gene conversion rates. 

## Results and discussion

**Figure 1:**
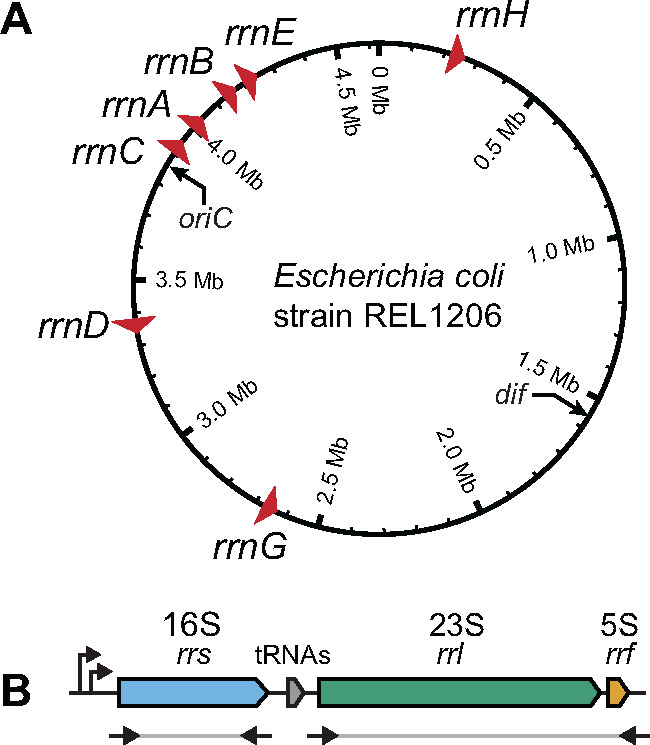
Ribosomal RNA operons in *E. coli*. (A) *Escherichia coli* B strain REL606 chromosome showing the locations and orientations of the seven rRNA operons. (B) Organization of a typical rRNA operon showing the two PCR amplicons that were sequenced at heterologous sites in evolved genomes isolated at the end of a 550-day mutation accumulation experiment.

**Figure 2:**
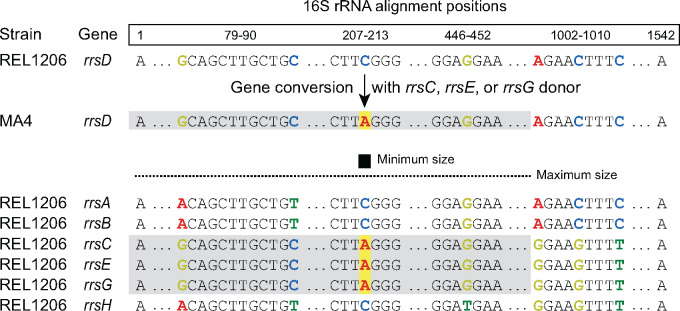
Example of a 16S rRNA gene conversion. In the sequenced endpoint clone from line 4 of the mutation accumulation experiment, a change of a C to an A was observed at position 210 of the 16S subunit alignment in the *rrnD* operon. Either the *rrnC*, the *rrnE*, or the *rrnG* operon 16S sequence could have acted as a donor to cause this change through a gene conversion. Because the sequence of *rrnD* already matched all three of these donors at other alignment positions from 1 to 1001, the actual gene conversion could have been as large as 1001 base pairs.

The *E. coli* chromosome encodes seven ribosomal operons, with a majority located near the origin of replication (Figure 1A). We sequenced amplicons corresponding to the 16S (*rrs*) and 23S + 5S (*rrl*+*rrf*) portions of each rRNA operon (Figure 1B) in endpoint clones from 15 independently evolved *E. coli* lineages from a 550-day MA experiment. We identified a total of 56 base substitutions and insertions or deletions of a few bases in the 16S and 23S genes of the evolved strains. All of these changes could be explained by gene conversions (as shown for one example in Figure 2). We did not find any new alleles in the rRNA genes that would require *de novo* point mutations to explain. We also observed four conversions that switched the 23S–5S intergenic region between a 186-bp sequence that is initially present in four of the rRNA operons and a shorter 92-bp variant present in the other three.

**Figure 3:**
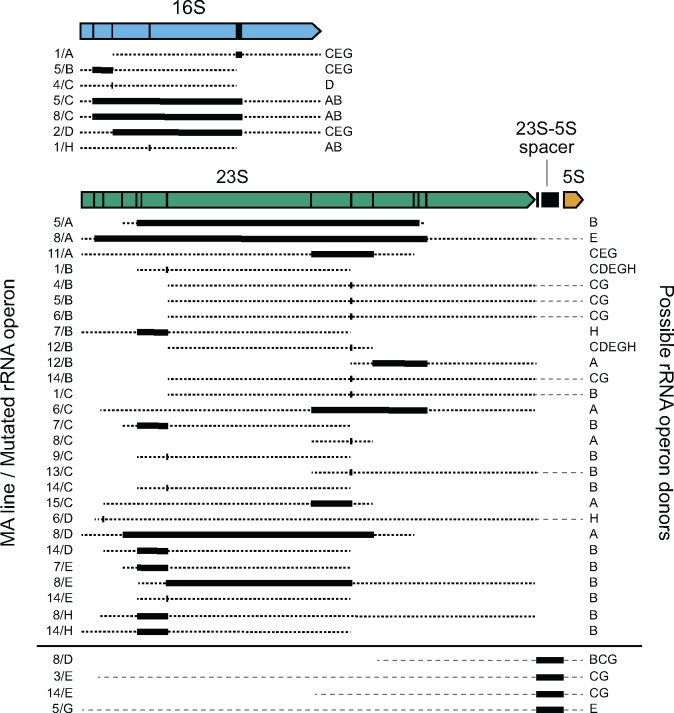
Gene conversions observed in rRNA operons during a mutation accumulation experiment. Bars in the genetic map indicate the locations of sequence differences between rRNA operons in the ancestral *E. coli* strain. In the remainder of the figure, boxes show the minimum possible extent of each conversion, and thick dotted lines show the maximum possible extent of each conversion. Thin dashed lines are used to indicate possible maximal extents that cross into or out of the 23S–5S spacer because conversions in this region were analyzed separately from conversions that could be localized within the 23S gene. Possible donors are listed for the largest gene conversion events that could have resulted in only the observed sequence changes; other donors may have been possible for smaller conversions.

The most parsimonious model (*i.e.* the fewest mutational events) that can reproduce the evolved rRNA sequences requires 38 gene conversions: 7 in the 16S subunit, 27 in the 23S subunit, and 4 in the 23S–5S spacer (Figure 3, Supplementary Table S1). These conversions change anywhere from a single base to all bases that differ between two rRNA copies within a contiguous 2137-bp region. We calculated conversion rates of 6.5 × 10^−5^ (95% confidence interval: 2.3 × 10^−5^ to 1.3 × 10^−4^) and 3.0 × 10^−4^ (95% confidence interval: 1.9 × 10^−4^ to 4.5 × 10^−4^) per genome in the 16S and 23S genes, respectively. Though there is some evidence that these rates are different from one another (likelihood ratio test, *P *=* *0.029), they were similar enough that we combined these observations to calculate an overall rate for these conversions, which all involve changing a small number of bases within mostly homologous sequences, of 3.6 × 10^−4^ per genome per generation (95% confidence interval: 2.6 × 10^−4^ to 4.7 × 10^−4^). This corresponds to a rate of 8.6 × 10^−6^ per rRNA operon per homologous donor operon per generation. From the MA experiment observations, we can also estimate that conversions covering the 23S–5S intergenic spacer region, which can swap its sequence between the two divergent alleles found in *E. coli*, occur at a rate of 3.5 × 10^−5^ per genome per generation (95% confidence interval: 9.0 × 10^−6^ to 9.1 × 10^−5^) or 8.3 × 10^−7^ per operon per donor per generation. This rate is similar to the one found for conversions within the 16S subunit, but somewhat lower than that found in 23S, which could be due to the smaller size of this region, the greater level of sequence divergence in the 23S–5S spacer than for any locus within these rRNA genes, or both.

A previous study of *E. coli* K-12 examined conversions that restored growth rates that had been compromised by removing a tRNA gene encoded in the 16S–23S spacer from all but one rRNA operon (Harvey and Hill 1990). It found that mutants, which propagated the spacer from the one remaining tRNA-containing rRNA operon to one of the other six operons through a gene conversion, appeared with a frequency of 6 × 10^−5^ per generation. Although this experimental design conflated population growth and mutation rates, the mutant frequency of 1 × 10^−6^ per recipient operon per donor per generation calculated from their results is surprisingly close to our estimate that conversions involving the 23S–5S spacer occur at a rate of 8.3 × 10^−7^ per operon per donor per generation. Another study found a conversion rate of 8 × 10^−10^ per operon per donor per generation in an *E. coli* B strain (Hashimoto *et al.* 2003). The very large deviation from our estimates (∼10^3^–10^4^ times less frequent) is likely due to their use of counterselection against a *sacB*-*neo* cassette inserted into the *rrsB* gene as a means for recovering mutants. Insertion of this large (3825 bp) non-homologous region is expected to introduce a substantial barrier to conversion, as gene conversion rates fall sharply with reduced homology (Morris and Drouin 2007).

Here, we specifically examined allelic gene conversions between rRNA operons. To quantify the rates of ectopic conversions between homologous genes, conversions between variants of *tufA* and *tufB* in *Salmonella typhimurium* that give rise to antibiotic resistance have been analyzed in a previous study (Abdulkarim and Hughes 1996). These genes share 98.9% sequence identity (1169/1182 bp) and experienced conversions at a rate of 2 × 10^−8^ per generation, which is ∼400 times less frequent than our overall 16S and 23S allelic conversion rate, even after adjusting for the 42 recipient–donor pairs that are possible for our rRNA conversions *vs* the one pair they studied. The 23S and 16S rRNA genes of different operons in *E. coli* are more similar, with an average sequence identity of 99.6%, which likely accounts for this difference.

**Figure 4:**
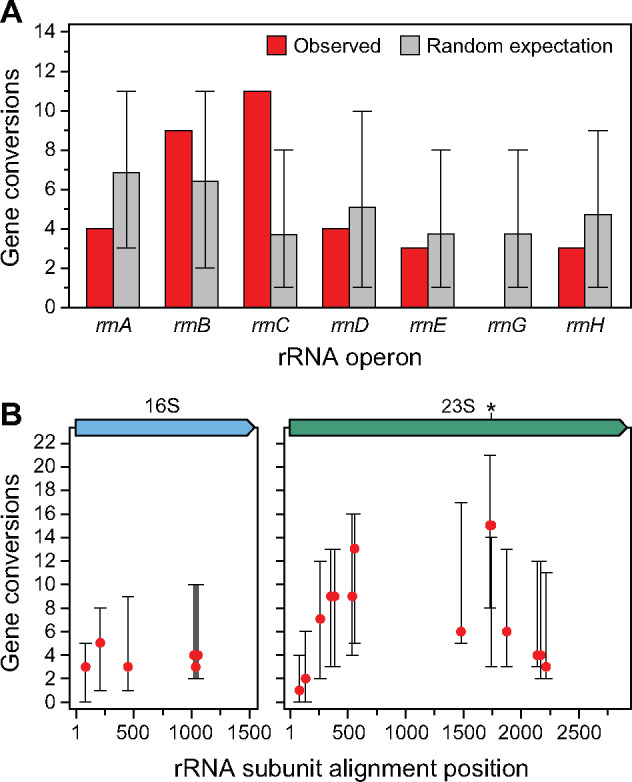
Distributions of conversion events changing the sequences of (A) rRNA operons and (B) heterologous sites within 16S and 23S rRNA genes. The random expectation values in A and error bars representing 95% confidence intervals in both A and B were estimated from 10,000 bootstrap resamplings of simulated gene conversions resembling those that were observed (see *Materials and methods*). The location of the Chi-like site in the 23S subunit in *rrnA* is starred.

We next examined whether certain rRNA operons were more likely to be converted than expected by chance (Figure 4A). Of the 34 observed gene conversions within the 16S or 23S subunits, 4 were identified in *rrnA*, 9 in *rrnB*, 11 in *rrnC*, 4 in *rrnD*, 3 in *rrnE*, and 3 in *rrnH*. No gene conversions were found in *rrnG*. Most operons showed no evidence of rate variation (adjusted *P *>* *0.05). The *rrnC* operon was the only exception: it was converted significantly more frequently than expected (adjusted *P *=* *0.013). Genes located near the *E. coli* origin of replication are copied first. Therefore, they exist transiently in two daughter chromosome copies for longer during the cell cycle compared to genes located further from the origin (Touchon *et al.* 2009). Additionally, rapidly growing bacteria can begin additional rounds of DNA replication before cell division is complete, resulting in even more copies per cell of genes that are located near the origin (Wang and Levin 2009). As *rrnC* is the closest rRNA operon to the *E. coli* origin of replication (Figure 1A), its elevated rate of gene conversions could reflect the higher effective copy number of this operon per cell compared to the other rRNA operons. The *rrnC* operon is also located in close proximity to three other rRNA operons (*rrnA, rrnB*, and *rrnD*), which is expected to increase the chances that it will undergo homologous recombination with those operons (Hashimoto *et al.* 2003). In fact, all 10 gene conversions we observed in the *rrnC* operon converted its sequence to match one of these three nearby operons, and 9 of the 10 converted it to match one of the two of these that are closest in the chromosome (*rrnA* or *rrnB*).

We also examined whether there was variation in the rates of conversions at certain sites within the 16S and 23S rRNA genes (Figure 4B). We did not find strong evidence that any heterologous sites were converted significantly more or less frequently than expected (adjusted *P *>* *0.05 for all sites). However, we did notice that sites at one locus in the 23S subunit were converted 15 times in the MA experiment and 2.5-fold more frequently than nearby sites ∼200 bp away (Figure 4B). The sequence at these sites is identical in six of the seven rRNA operons. However, in the seventh operon (*rrnA*) this site contains a sequence (5′-GCTCGTGG-3′) that differs by one base from a canonical *E. coli* Chi site (5′-GCTGGTGG-3′). Similar Chi-like sites retain up to 40% of the activity of a consensus Chi site in promoting homologous recombination (Smith 2012), which suggests that this sequence may be responsible for the somewhat elevated gene conversion rate observed at this location. In support of this hypothesis, *rrnA* was the donor or recipient for 7 of the 15 conversions involving this site.

**Figure 5:**
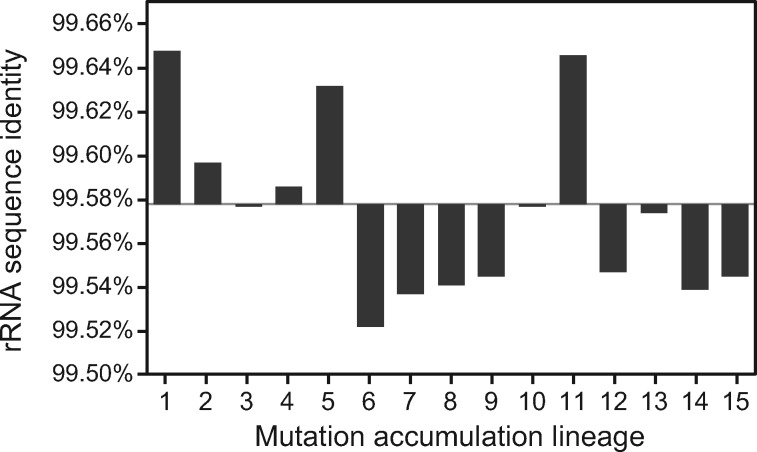
Evolution of average pairwise sequence identity in 23S and 16S rRNA subunits during the mutation accumulation experiment. The final value in each evolved lineage is depicted as a bar indicating the change from the 99.578% rRNA identity present in the ancestral *E. coli* strain.

Gene conversions maintain homogeneity between rRNA operons on long evolutionary timescales (Liao 2000; Hashimoto *et al.* 2003), and we wondered whether the sequences of the rRNA operons also tended to become less distinct from one another on the relatively short evolutionary timescale of the MA experiment. Considered together, the 16S and 23S sequences in the seven rRNA operons have an average pairwise sequence identity of 99.578% in the ancestor of the MA experiment. The change in this identity ranged from +0.070% to −0.055% in the 15 MA lineages (Figure 5), with no significant overall trend up or down (Wilcoxon signed rank test, *P *=* *0.45). Sequence identity is only irreversibly ratcheted up by gene conversions when they completely eliminate an alternative allele from all copies of a gene, and the modest number of conversions that we observed was not enough to achieve this during the MA experiment. Therefore, this result underscores that homogenization via conversion is likely to only take place on long timescales, particularly for sequences with many copies like the *E. coli* rRNA operons.

The spontaneous rate of base substitution mutations in *E. coli* is ∼10^−10^ per base pair per generation (Wielgoss *et al.* 2011; Foster *et al.* 2015). It follows that the rate at which new base changes accumulate in the 23S and 16S genes in its seven rRNA operons (∼30,000 total base pairs) is at most ∼3 × 10^−6^ per genome per generation and probably less than this because many of these mutations will be deleterious. We found a gene conversion rate of 3.6 × 10^−4^ per genome per generation, which is greater by at least 2 orders of magnitude. This difference between conversion and mutation rates is expected to contribute to the concerted evolution of rRNA sequences in the long term. New point mutations that could increase within-genome rRNA sequence diversity in *E. coli* are expected to be reverted or propagated to all rRNA copies by the many (>100 or more) gene conversions that will occur in a lineage for every point mutation.

It is especially important to understand the relative balance and timescales of these mutational processes because rRNA sequences are used for phylogenetic reconstruction and metagenomic community profiling. Others have noted how heterogeneity in rRNA operons within a genome can complicate these analyses, registering individual species as separate taxa (Ionescu *et al.* 2017) and overestimating the number of different taxa within a community (Dahllöf *et al.* 2000). Gene conversions can also obscure evidence of rRNA horizontal gene transfer when an acquired copy is homogenized (Tian *et al.* 2015) or confuse phylogenetic placement when native rRNA genes are converted to match an acquired copy (Hao and Palmer 2011). Gene conversions have even been observed on short timescales during routine propagation of strains in the laboratory, including a conversion in the *rrlH* gene of the *E. coli* REL606 strain from which the ancestor of our MA lineages evolved (Studier *et al.* 2009). More broadly, conversion rates influence the chances that paralogs of any gene can evolve sequence diversity and stably maintain distinct functions after gene duplication (Teshima and Innan 2008). Our measurements of *E. coli* rRNA gene conversion rates enable more accurate modeling of these processes and improve our overall understanding of bacterial genome evolution.

## Funding

This work was supported by the Welch Foundation (Grant No. F-1979) and the US Army Research Office (Grant No. W911NF-12-1-0390). 

*Conflicts of interest:* None declared.
